# Manual Therapy-Associated Dural Tear Causing Intracranial Hypotension Treated with an Epidural Blood Patch: A Case Report

**DOI:** 10.3390/jcm15103860

**Published:** 2026-05-17

**Authors:** Niklavs Nemme, Arturs Balodis, Mara Klibus, Olegs Sabelnikovs, Arina Novasa, Jolanta Osina, Marina Sarkele

**Affiliations:** 1 Department of Anaesthesiology, Intensive Care and Clinical Simulations, Riga Stradins University, LV-1007 Riga, Latvia; mara.klibus@gmail.com (M.K.); olegs.sabelnikovs@rsu.lv (O.S.); marina.sarkele@rsu.lv (M.S.); 2 Faculty of Medicine, Riga Stradins University, LV-1007 Riga, Latvia; 3 Department of Radiology, Riga Stradins University, LV-1007 Riga, Latvia; arturs.balodis@rsu.lv; 4 Institute of Diagnostic Radiology, Paula Stradins Clinical University Hospital, LV-1002 Riga, Latvia; 5 Department of Anaesthesiology, Pauls Stradins Clinical University Hospital, LV-1002 Riga, Latvia; 6 Department of Neurology, Pauls Stradins Clinical University Hospital, LV-1002 Riga, Latvia; arina.novasa@stradini.lv (A.N.); jolanta.osina@gmail.com (J.O.); 7 Residency in Algology, Faculty of Residency, Riga Stradins University, LV-1007 Riga, Latvia

**Keywords:** intracranial hypotension, dural tear, epidural blood patch, brain sagging, subdural hematoma

## Abstract

**Background/Objectives**: Intracranial hypotension is a rare and underdiagnosed serious condition characterized by low cerebrospinal fluid (CSF) pressure, often resulting from trauma to the dura mater. While manual therapy is increasingly used for musculoskeletal complaints, it is not without risk and may, in rare cases, result in complications such as dural tears. Although these complications are rare, they require early recognition and appropriate treatment to prevent further morbidity. This case report aims to highlight a rare presentation of multilevel dural defects in temporal association with manual therapy and to demonstrate the efficacy of epidural blood patch (EBP) treatment. **Case Presentation**: We report a case of a 46-year-old woman without chronic illness who developed worsening orthostatic headaches, weakness, and vomiting after multiple manual therapy sessions. Only after 6 months did the patient undergo magnetic resonance imaging (MRI), which revealed intracranial hypotension due to dural damage in the spinal dura mater at C6–T1 and T8–T10, brain sagging, and an increased risk of subdural hematoma. After excluding other causes of dural defects, EBP was performed under CT guidance at C6–C7 and T8–T9, which resulted in symptom regression. Follow-up MRI was recommended for the patient. **Conclusions**: This case highlights a rare but clinically significant occurrence of multilevel dural defects and intracranial hypotension in temporal association with manual therapy. This emphasizes the critical role of timely diagnosis using MRI and the clinical effectiveness of EBP as a minimally invasive procedure.

## 1. Introduction

Normal cerebrospinal fluid (CSF) pressure is approximately 60 to 200 mm of H_2_O, and intracranial hypotension (IH) is defined as a CSF pressure of less than 60 mm H_2_O [1,2].

The etiology of IH can be divided into two main categories: spontaneous and iatrogenic. While iatrogenic IH is typically associated with spinal taps, anesthesia, and spinal surgery, spontaneous IH can be linked to disc spurs, osteophytes, and connective tissue disorders such as Marfan syndrome, neurofibromatosis, and Ehlers–Danlos syndrome. Additionally, minor trauma, such as twisting or stretching of the meninges, can be associated with ruptures of underlying spinal epidural cysts or tears in the dural nerve sheath. While the most common cause of CSF leaks are meningeal diverticula, the second most common cause is dural tears [2,3].

The usual symptoms that patients complain about are headaches, specifically orthostatic headaches, which typically improve in a recumbent position. Occasionally, patients may complain of nausea, dizziness, vomiting, neck pain and stiffness, photophobia, diplopia, and other symptoms, which can develop into stupor or coma. The onset of these symptoms may vary from patient to patient, ranging from 15 min to even 2 or more hours later after the dural tear [1,2,3].

IH diagnosis is based on the patient’s medical history, clinical features, and diagnostic radiology. Unfortunately, due to inadequate awareness of IH, the diagnostic delay after the onset of symptoms may be an average of 13 months. The International Classification of Headache Disorders (ICHD-3) states that the diagnosis of spontaneous IH requires the presence of a headache that is related to low CSF pressure or CSF leakage and is confirmed by either low CSF pressure (<60 mm H_2_O) or imaging evidence of CSF leakage; additionally, it must not be better explained by any other headache disorder. If left undiagnosed and untreated, it increases the risk of brain herniation and stroke, thereby increasing morbidity [1,4,5].

MRI is the diagnostic tool of choice for IH, demonstrating enhancement of the pachymeninges, downward displacement of the brain, and subdural fluid collections in most patients. Additionally, CT myelography can be used to identify the topography of CSF leaks [4,6].

Most patients with spontaneous IH are treated conservatively with bed rest, oral caffeine, oral hydration and mineralocorticoids and glucocorticoids. If conservative therapy fails, an epidural blood patch (EBP) is recommended. After an EBP procedure, patients typically show clinical improvement within the first 24 to 48 h, but radiological improvement can be delayed and variable. A recent series showed that patients treated either conservatively or with EBP revealed similar rates of improvement during one and six month follow-up MRIs. While EBPs can suddenly improve CSF pressure and close the tear due to the injected autologous blood, radiological signs such as meningeal thickening, subdural collections, and engorgement of the pituitary gland may resolve as late as 6 months. Therefore, several studies have emphasized that the outcome is primarily based on clinical signs, since MRI signs may persist for months after treatment. Commonly used criteria for the evaluation of treatment success include at least a 50% reduction in the headache and orthostatic component, at discharge as well as three months after discharge [1,7,8,9,10,11,12].

The goal of manual therapy is to lessen the burden of global disability by targeting musculoskeletal conditions in fields such as physiotherapy, osteopathy and chiropractic. While benign adverse events can be common, typically mild, and transient, such as localized soreness and fatigue, serious adverse events are described as rare but may include vertebral injury, cervical artery dissection, dural tears, and other complications that require medical attention [13,14,15].

This case report aims to highlight the rare and underdiagnosed multilevel dural defects following manual therapy leading to intracranial hypotension, and to demonstrate the efficacy of EBP treatment. Additionally, this report underscores the importance of awareness among clinicians regarding this complication of manual therapy and its management to improve patient outcomes.

## 2. Case Report

We report a case of a 46-year-old woman without chronic illness or drug allergies who developed throbbing headaches and a stiff neck upon standing the following morning, approximately 12 h after a manual therapy session. The manual therapy session included spinal manipulation involving high-velocity and low-amplitude thrusts applied to the joints of the spine. The orthostatic headache persisted for a week, and only the supine position provided some relief to the patient. The next day, the patient developed nausea and vomiting and was admitted to a regional hospital. Contrast-enhanced CT (a 64-slice computed tomography scanner, Revolution Maxima (GE HealthCare, Chicago, IL, USA), was used) of the head with an angiogram of the brachiocephalic vasculature was performed, but they did not reveal any abnormalities. Additionally, a lumbar puncture was performed, however the procedure yielded a low CSF volume. Neurological examination revealed no abnormal findings, and a diagnosis of aseptic meningitis was made. The symptoms subsided within 3 days of bed rest and fluid intake, and the patient was discharged from the hospital.

After returning home, the headaches reappeared a few days later but with mild intensity, thus, no action was taken and the headaches subsided completely.

After four months, the patient started to experience the same headaches as before; in addition, rapid movements—such as riding a bike over uneven surfaces—caused immense pain. The patient reported being under prolonged high stress at that time and did not consult a medical professional for another month. Although the patient reported taking non-steroidal anti-inflammatory drugs, no significant benefit was observed. The following month, she was admitted to a tertiary care center.

On examination, the patient was hemodynamically stable and verbally responsive. Her body mass index was 31.6 kg/m^2^. The physical examination findings were unremarkable. Neurological examination revealed no abnormalities.

**Figure 1:**
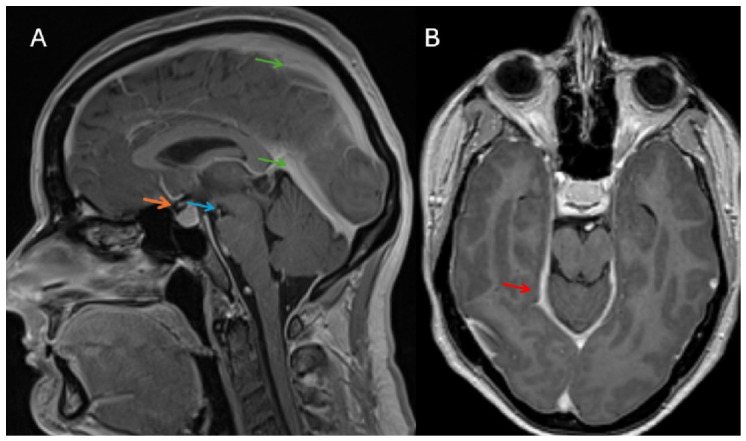
Post-contrast T1-weighted MRI images in sagittal (**A**) and axial (**B**) planes obtained after intravenous gadolinium administration. The images demonstrate multiple signs of intracranial hypotension. The red arrow indicates diffuse, smooth pachymeningeal enhancement. The orange arrow shows pituitary enlargement due to venous engorgement. The blue arrow marks reduced size of the suprasellar and prepontine cisterns. The green arrow highlights engorged venous sinuses. Image (**B**) shows a decreased ventricular size (not indicated by an arrow).

Repeated brain and spine MRI (magnetic resonance imaging was performed using a MAGNETOM Avanto 1.5 T scanner (Siemens Healthineers, Erlangen, Germany) revealed IH signs, such as diffuse and smooth pachymeningeal enhancement and pituitary enlargement (Figure 1).

**Figure 2:**
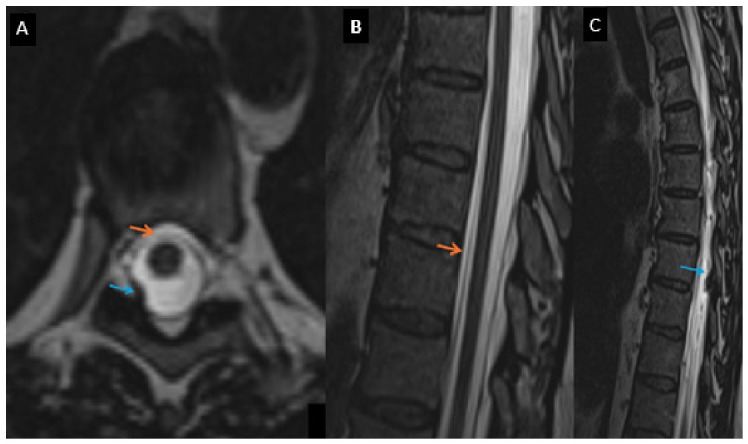
High-resolution T2-weighted SPACE (Sampling Perfection with Application-optimized Contrasts using different flip-angle Evolutions) iso-voxel thin-slice images (0.9 mm) in axial and sagittal planes (**A**–**C**). The orange arrow indicates a ventral epidural fluid collection. The blue arrow marks a T8 osteophyte arising from the intervertebral joint, causing indentation of the dural sac and representing the suspected site of a dural defect, which is better visualized in image F.

Spinal MRI indicated a ventral epidural fluid collection and the potential localization of a dural defect at the T8 level (Figure 2).

**Figure 3:**
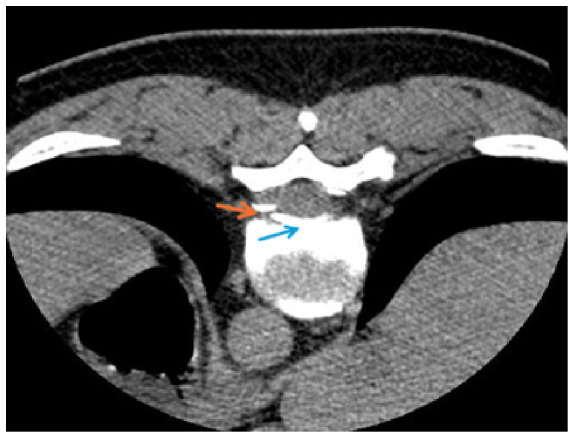
CT myelography image obtained after intrathecal administration of iodinated contrast material (blue arrow indicates intrathecal contrast). At the T8 level, there is focal contrast extravasation through a dural defect (orange arrow), with leakage into the ventral epidural space between the dural layers, confirming the site of the cerebrospinal fluid (CSF) leak.

An additional CT myelography scan verified signs of CSF leakage at the T8 level (Figure 3).

High-resolution T2-weighted SPACE MRI revealed an osteophyte at the T8 level, suggesting a potential focal site of dural disruption. At the C6–T1 level, epidural fluid collection posterior to the dura mater was observed without direct visualization of a discrete dural defect on 3D T2-weighted images. Given that epidural fluid may extend over multiple spinal levels from a single leak site, these findings were interpreted with caution and do not establish an independent site of CSF leakage. Based on these findings, a targeted epidural blood patch (EBP) was performed at the T8–T9 level, and an additional preventive EBP was applied at C6–C7. Both procedures were performed under CT guidance.

Epidural blood patch (EBP) procedures were performed under CT guidance using a standard sterile technique. The patient was placed in the prone position, and the target interlaminar space was identified. After skin antisepsis and local anesthesia, a Tuohy needle was advanced into the epidural space using imaging guidance and confirmation of appropriate positioning. Autologous venous blood was then aseptically collected and slowly injected into the epidural space. The injection was performed incrementally, with careful monitoring of patient-reported pressure or discomfort, which guided the total injected volume. The procedure was terminated upon the onset of significant pressure sensation or when the planned volume had been administered. Following the procedure, the patient remained in the supine position for observation and was monitored for symptom improvement and potential complications.

The patient tolerated the procedure well, and the initial symptoms subdued. She experienced mild residual pain at the EBP site, which decreased progressively each day. No neurological abnormalities were observed.

After discharge, the patient was advised to maintain bed rest for 10 days, followed by a gradual increase in upright activity, adequate hydration (2.5 L/24 h), no lifting of heavy objects, manual therapy, and gymnastics for 1 month. The patient was advised to avoid excessive neck flexion and extension. Pharmacological therapy included tab. tolperisonum 150 mg BID for 2 weeks, cap. gabapentinum 300 mg qHS for 1 month, tab. naproxenum 550 mg for pain and tab. theophyllinum 200 mg BID for 3 weeks. The patient was scheduled for brain and spine MRI without contrast after one month.

During the control visit in September 2025, brain and spine MRI without contrast revealed minor improvement, and intracranial hypotension was still present with dural defects. The patient reported transient headaches in response to stress, with a pain intensity of 3/10 on the visual analog scale for pain. Additionally, fatigue and weakness improved but remained persistent. The recommendations remained unchanged; however, physical activity was restricted for three months instead of one month. The patient was also advised to consume caffeinated beverages, and a follow-up MRI without contrast was scheduled in three months. The patient continues to be monitored by her general practitioner.

**Figure 4:**
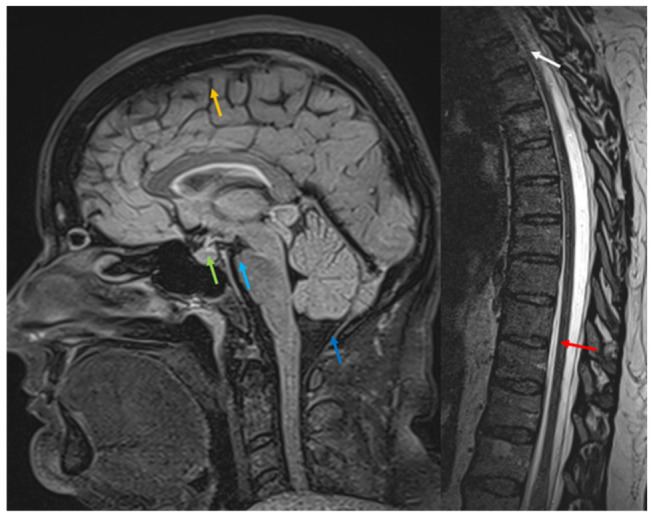
Six-month follow-up MRI after the procedure. Sagittal 3D FLAIR and sagittal T2 SPACE (0.8 mm) images are shown. The yellow arrow indicates that no further effusion or dural thickening is visible on follow-up imaging. The green arrow demonstrates resolution of previous pituitary gland enlargement, with improved visualization of the infundibulum and normal appearance of the pontomammillary distance (light blue arrow). The dark blue arrow shows no longer visible narrowing of the craniocervical junction, with no radiological signs of intracranial hypertension. On the T2 SPACE sagittal image, the previously noted thin dural double contour at the T8–T9 level (red arrow) demonstrates a reduction in the inter-dural content on follow-up imaging. At the cervicothoracic junction (C7–T1, white arrow), the dural duplication is no longer visible.

MRI performed during follow-up demonstrated IH regression 6 months after EBP. Compared with the previous MRI, the pontomammillary distance had increased and was approaching the lower limit of normal. The basal cisterns were differentiable, the pituitary gland had returned to its normal size, and no subdural effusions were present (Figure 4).

## 3. Discussion

This case report describes a rare presentation of multilevel defects associated with intracranial hypotension (IH) following manual therapy and demonstrates the clinical effectiveness of targeted epidural blood patch (EBP) treatment. Although IH is most commonly linked to spontaneous leaks or recognized iatrogenic causes such as lumbar puncture and spinal surgery [2], this case supports the accumulating evidence that mechanical spinal manipulation may act as a precipitating factor in susceptible individuals.

The existing literature on spinal manual therapy suggests that some practitioners performing these interventions may not consistently communicate the potential risks of serious adverse events to patients. Although there is agreement that risk disclosure is the moral and ethical thing to do, there is concern among professionals that it may increase patient anxiety and possible refusal of care [13].

A review of the available literature indicates that intracranial hypotension associated with spinal manual therapy is rare and is primarily described in isolated case reports rather than in large observational studies. In the broader context, spontaneous intracranial hypotension is estimated to occur in approximately 5 per 100,000 people annually. In the largest observational study of 568 patients with spontaneous CSF leaks, the patients were categorized into single ventral or lateral dural tears, meningeal diverticula, or CSF-venous fistulas. It was observed that multilevel leaks occurred only in 6% of patients with lateral leaks and 9% of patients with CSF-venous fistulas. Reported cases involve a range of techniques, including chiropractic manipulation, osteopathic treatment, and physiotherapy-based mobilization. The mentioned techniques can produce brief but substantial multidirectional loads and with vertebral motion and complex force patterns can be transferred across the segment in motion, illustrating possible pathways for focal stress concentration on the ventral dura. In the presence of cervical rotatory manipulation, canal compromise significantly increases von Mises stress in the dura mater, spinal cord, and nerve roots. Imaging findings range from clearly identifiable dural defects to indirect signs, such as epidural fluid collections without a precisely localized leak site. The underlying mechanisms are thought to be multifactorial, including direct mechanical stress on the dura, exacerbation of pre-existing structural weaknesses, or interaction with degenerative spinal changes such as osteophytes or disc pathology. As summarized in Table 1, these reports highlight the heterogeneity of presentations and, in some cases, the challenges in definitively identifying the site of leakage. Importantly, although such complications are rare, they underscore the need for awareness of potential adverse events associated with spinal manual therapy and the importance of careful clinical evaluation and appropriate patient counseling [16,17,18,19,20,21,22].

The patient had a body mass index (BMI) of 31.6 kg/m^2^. In individuals with an elevated BMI, greater force may be required during manual therapy to achieve the desired therapeutic effect, potentially increasing mechanical stress on spinal structures. However, there is currently no direct evidence linking elevated BMI to an increased risk of dural injury. Therefore, a causal contribution of increased biomechanical load to the dura cannot be established and remains speculative.

There were no clinical features suggestive of an underlying connective tissue disorder, such as joint hypermobility or skin hyperextensibility. No evidence of a condition such as Ehlers–Danlos syndrome or Marfan syndrome was identified on clinical examination. Connective tissue disorders have been reported as potential predisposing factors for spontaneous cerebrospinal fluid (CSF) leaks due to structural dural weakness [23]; however, such features were not observed in this patient. Although no clinical suspicion of an underlying disorder was present, the possibility of subclinical connective tissue vulnerability cannot be entirely excluded.

The patient reported independently seeking manual therapy for preventive care. She did not have any prior radiological imaging of the brain and spine to evaluate the anatomical structures before manual therapy of the spine.

IH results from reduced CSF volume and pressure, most frequently due to spinal CSF leakage. The hallmark clinical feature is orthostatic headache, often accompanied by nausea, vomiting, and neck pain; furthermore, other neurological symptoms have been reported, including dizziness, tinnitus, hearing disturbances, visual symptoms (such as diplopia or blurred vision), cognitive changes, and, less commonly, cranial nerve deficits or radicular symptoms. These manifestations reflect the diverse clinical presentation of intracranial hypotension and may vary depending on the severity and duration of CSF leakage [1,2]. In the present case, the patient exhibited classic orthostatic symptoms shortly after manual therapy, yet the initial diagnosis was aseptic meningitis. This reflects the well-documented diagnostic challenge of IH and the tendency for delayed recognition, particularly when recent invasive procedures are absent [4].

Manual therapy is widely used in physiotherapy, chiropractic, and osteopathic practices and is generally considered safe. Most reported adverse events were mild and transient. However, serious complications, including cervical artery dissection, vertebral injury, and dural tears, have been described, albeit rarely [13]. The exact mechanism by which manual therapy may lead to dural disruption remains unclear [24]. Proposed mechanisms include excessive rotational or traction forces transmitted to the dura, sudden changes in intraspinal pressure, or rupture of pre-existing meningeal weaknesses, such as diverticula [13,25]. In the current patient, no alternative etiologies for CSF leakage were identified, and the temporal relationship between repeated manual therapy sessions and symptom onset supports a probable association. Nevertheless, causality cannot be definitively established.

The identification of an osteophyte at the T8 level provides a plausible structural explanation for the observed CSF leak. Ventral dural tears related to degenerative spinal changes, particularly osteophytes or calcified intervertebral disc pathology, are well described in the literature as a common mechanism of spontaneous intracranial hypotension [26]. These bony abnormalities may mechanically compromise the ventral dura, sometimes through focal protrusions or microspurs that can incise or weaken the dural layer. In this context, the osteophyte observed in our case may have acted as a predisposing factor for focal dural vulnerability. Although a direct causal relationship cannot be definitively established, it is conceivable that additional mechanical stress, such as manual therapy, may have served as a trigger for dural disruption. This interpretation is consistent with previous reports suggesting that minor or trivial trauma may precipitate CSF leakage in the presence of underlying structural weakness [27].

In parallel, the patient’s elevated body mass index (31.6 kg/m^2^) may have contributed to increased mechanical loading of the spine during manual therapy. It is therefore plausible that these factors acted synergistically, with the osteophyte predisposing the dura to focal weakness and increased biomechanical stress, facilitating dural disruption. This combined effect may have heightened susceptibility to CSF leakage at this level.

A notable feature of this case is the presence of multilevel dural defects at C6–T1 and T8–T10 levels. Most reported CSF leaks involve a single spinal level, whereas multifocal leaks are rare [21]. This finding may suggest either the transmission of mechanical forces across multiple spinal segments during manipulation or an underlying predisposition to dural fragility.

Spinal MRI plays a central role in evaluating cerebrospinal fluid (CSF) leakage in spontaneous intracranial hypotension. In addition to identifying epidural fluid collections, several characteristic imaging signs have been described. One such feature is the “floating dural sac sign,” which reflects circumferential epidural CSF accumulation resulting in separation and inward displacement of the dural sac. The patient’s T2-weighted MRI sequences demonstrate the above-mentioned “floating dural sac sign”. This sign is considered a useful indicator of significant epidural fluid presence and may aid in the diagnosis of CSF leakage, even when the exact site of dural disruption is not directly visualized. The recognition of such indirect imaging findings can improve diagnostic confidence and facilitate appropriate management [28].

The case emphasizes the importance of appropriate imaging. Early CT and clinical assessments were insufficient to establish a diagnosis. MRI remains the diagnostic modality of choice for IH, with typical findings including pachymeningeal enhancement, brain sagging, venous engorgement, and extradural fluid collections [4,11]. In this patient, a repeat brain and spine MRI was decisive in identifying both intracranial features of hypotension and specific dural defect sites. Clinicians should maintain a high index of suspicion and consider early MRI in patients presenting with new-onset orthostatic headache following spinal manipulation [1].

Untreated IH may lead to serious complications, including subdural hematoma, cranial nerve palsies, and, in severe cases, brain herniation [11]. Brain sagging observed in this patient indicated significant CSF depletion and justified active intervention. Current management strategies recommend initial conservative therapy; however, when symptoms persist, or imaging demonstrates a clear leak, EBP is considered the treatment of choice [1,4,11].

EBP is performed by administering autologous blood into the epidural space. While lumbar EBP does not always require computed tomography (CT) control, thoracic and cervical EBPs are recommended to be performed in combination with fluoroscopy or CT scan guidance to optimally cover the region of interest and reduce the risk of complications. Although the patient can be placed in a seated, lateral, or prone (if fluoroscopic guidance is used) position for the procedure, it is essential to consider the patient’s tolerance and procedural considerations. After an average dose of 30 mL injection of autologous blood or the patient developing back pain, the procedure is stopped, and the patient is placed in a supine position for up to 2 h to allow the blood to spread to other spinal levels. In most patients, the CSF leak is located in the thoracic or cervical spine, with one study reporting that 72% of CSF leaks occur at the thoracic level, often due to dura mater tears [1,4,11,12,29].

Some patients may require EBP to be performed at multiple levels to maximize the coverage of the CSF leak site. When it comes to spontaneous IH and EBP, higher amounts of injected autologous blood are associated with greater initial EBP efficacy. For this reason, multilevel EBP strategies can outperform single-level approaches. Data indicates that larger volumes of injections and more extensive craniocaudal distribution are the main determinants of success, especially for multilevel leaks [10,30,31].

While EBP is successful in 30–70% of spontaneous IH patients, in cases where it has failed, additional treatment interventions may be performed such as repeated EBP, fibrin glue patch, and surgical repair. Fibrin glue patching is performed in the same manner as an EBP, either using fibrin glue instead of autologous blood or the combination of both. Surgery, on the other hand, is indicated in cases where the symptoms persist despite previous less invasive treatment and if the location of the leak is verified using radiological techniques. During surgery, the damaged location can be repaired using suturing or using adhesive patches [32,33].

EBP acts through both immediate and delayed mechanisms, including epidural tamponade and subsequent sealing of the dural defect via clot formation and fibrosis [12]. Targeted EBP at both identified levels in this case resulted in rapid clinical improvement within the first 24 h, consistent with reported success rates in the literature. A study has reported that for around 28.3% of women, the EBP failed, and a total of 19.8% received a second EBP due to the numeric rating scale being a score of 7 or more in the upright position at 4 for 24 to 48 h. The need for multilevel patching reflects the multifocal pathology and highlights the importance of precise leak localization. Persistent but improving radiological findings at follow-up are not uncommon and do not necessarily indicate treatment failure when clinical recovery is evident [11,12].

Overall, the available literature shows associations but not definitive causality between specific mechanisms and multilevel dural defects or CSF leaks. Factors such as small sample sizes, incomplete patient histories, confounding factors, and misclassification all substantially restrict causal association about both the etiology and frequency [17,23,34,35].

From a clinical practice perspective, this case underscores several important considerations. First, although rare, serious complications of manual therapy can occur and should be promptly recognized. Second, orthostatic headache after spinal manipulation warrants careful evaluation for IH. Third, MRI of both the brain and spine is essential for accurate diagnosis and treatment planning. Finally, EBP represents an effective and minimally invasive therapeutic option, even for multilevel CSF leaks.

## 4. Conclusions

This case report highlights a rare but clinically significant occurrence of multilevel dural defects and intracranial hypotension in temporal association with manual therapy. Serious adverse events from spinal manipulation are uncommon. Clinicians should remain vigilant when patients present with new-onset orthostatic headaches after these interventions. Early recognition and use of appropriate imaging—especially MRI of the brain and spine—is essential. Prompt action helps to avoid diagnostic delay and complications, including subdural hematoma.

Targeted EBP proved to be an effective and minimally invasive treatment for this patient, resulting in marked clinical improvement. This case underscores the importance of maintaining clinical suspicion for CSF leakage in atypical headache presentations and supports the role of EBP as a key therapeutic option. Increased awareness among healthcare providers and clear communication of the potential risks associated with manual therapy may contribute to earlier diagnosis and improved patient outcomes.

## Figures and Tables

**Table 1:** Summary of previously reported cases of intracranial hypotension associated with spinal manual therapy.

Author (Year)	Type of Manual Therapy	Spinal Level Involved	Imaging Findings	Proposed Mechanism	Outcome
Bozer (2024) [17]	Chiropractic thoracic manipulation	Thoracic	Diffuse epidural CSF collection,	Dural tear following chiropractic spinal manipulation therapy	Improvement after epidural blood patch
J. Sutton (2022) [19]	Chiropractic cervical manipulation	Cervical	Spinal CSF leak at C7	Dural tear following chiropractic spinal manipulation	Improvement after epidural blood patch
Kusnezov. (2013) [16]	Chiropractic manipulation	Cervical	Epidural fluid collection, CSF leak C5–C6	Mechanical stress on dura	Symptom resolution after conservative treatment
E. Kim (2024) [20]	Cervical chiropraxis	Cervical	Upper cervical level	Mechanical strain at the level of the cervical root sleeves	Improvement after epidural blood patch

## Data Availability

The original contributions presented in this study are included in the article. Further inquiries can be directed at the corresponding author.

## Abbreviations

The following abbreviations are used in this manuscript:

IH	Intracranial hypotension
EBP	Epidural blood patch
CSF	Cerebrospinal fluid
CT	Computed tomography
MRI	Magnetic resonance imaging
